# Antibiotic saving effect of combination therapy through synergistic interactions between well-characterized chito-oligosaccharides and commercial antifungals against medically relevant yeasts

**DOI:** 10.1371/journal.pone.0227098

**Published:** 2019-12-31

**Authors:** Monica Ganan, Silje B. Lorentzen, Berit B. Aam, Vincent G. H. Eijsink, Peter Gaustad, Morten Sørlie

**Affiliations:** 1 Department of Chemistry, Biotechnology, and Food Science, Norwegian University of Life Sciences, Aas, Norway; 2 Institute of Clinical Medicine, Department of Microbiology, University of Oslo, Blindern, Oslo, Norway; University of South Carolina, UNITED STATES

## Abstract

Combination therapies can be a help to overcome resistance to current antifungals in humans. The combined activity of commercial antifungals and soluble and well-defined low molecular weight chitosan with average degrees of polymerization (DP_n_) of 17–62 (abbreviated C17 –C62) and fraction of acetylation (*F*_A_) of 0.15 against medically relevant yeast strains was studied. The minimal inhibitory concentration (MIC) of C32 varied greatly among strains, ranging from > 5000 μg mL^-1^ (*Candida albicans* and *C*. *glabrata*) to < 4.9 (*C*. *tropicalis*). A synergistic effect was observed between C32 and the different antifungals tested for most of the strains. Testing of several CHOS preparations indicated that the highest synergistic effects are obtained for fractions with a DP_n_ in the 30–50 range. Pre-exposure to C32 enhanced the antifungal effect of fluconazole and amphotericin B. A concentration-dependent post-antifungal effect conserved even 24 h after C32 removal was observed. The combination of C32 and commercial antifungals together or as part of a sequential therapy opens new therapeutic perspectives for treating yeast infections in humans.

## Introduction

With the increased competence of medical science to extend the lives of immunocompromised hosts, the incidence of systemic fungal infections has raised dramatically. For years, amphotericin B has been considered the “gold” standard for the treatment of invasive fungal infections, but toxicity limits its usefulness. Nowadays, less toxic triazole antifungals, such as fluconazole and itraconazole, are considered reasonable substitutes. However, in spite of amphotericin B or triazole monotherapy treatments, mortality associated with fungal infections remains to be substantial. There is a great interest in using combined therapies in an attempt of improving survival rates and of reducing fungal resistance. For this reason, a great number of studies have investigated the synergistic activity of commercial antifungals (CA) [1–4].

Antifungal resistance makes infections harder to treat and is an increasing problem. *Candida* spp are increasingly resistant to antifungal treatment with azoles and echinocandins. The most resistant *Candida* spp are *C*. *glabrata*, *C*. *krusei* and the emerging new species *C*. *auris* [5–7]. In less common fungal infections as with the mold *Aspergillus fumigatus*, emerging resistance to azoles threatens the effectiveness of life-saving medications. Resistant *Aspergillus* infections can develop in people who use antifungals and agricultural use of azole fungicides to treat crop diseases, lead to the growth of resistant strains of *Aspergillus* and people with weakened immune systems is at risk to be infected [8, 9]. Other molds as *Fusarium* spp. [10] and *Scedosporium* spp have been increasingly recognized as cause of resistant life-threatening infections. Treatment of filamentous fungi species, is particularly challenging because of their resistance to many antifungal agents [11].

Chitin is a polysaccharide that consists of β(1→4) linked N-acetyl-D-glucosamine residues. It is the second most abundant biopolymer in nature after cellulose, as it is an important component of the cell walls of fungi, and yeasts, and of the shells of insects and crustaceans. Chitosan is a cationic polymer obtained by the alkaline partial of full deacetylation of chitin. The use of oligomers of chitosan, known as chito-oligosaccharides or CHOS, is of major interest since CHOS have a variety of interesting biological activities and are more soluble than chitosan [12]. The number of monomeric units defines the polymerization degree of the CHOS (DP), and the fraction of acetylation (F_A_) refers to the average fraction of acetylated monomers (GlcNAc units). These two features determine important physical-chemical properties of the CHOS, like solubility and conformation [13].

In recent years, chitosan and CHOS have received remarkable attention due to their potential use in medicine, and since they are considered to be biodegradable, non-toxic, non-immunogenic and non-carcinogenic. Chitosan has been proposed as delivery system for different antifungals, including amphotericin B [14, 15] and fluconazole [16, 17]. Moreover, the polymer has a well-documented antifungal activity itself [18–20]. Recently, we demonstrated antifungal activity of well-defined chito-oligosaccharide preparations against medically relevant yeasts [21]. The aim of the work presented in this report is to study the combined antifungal pharmacodynamics of a these well-characterized CHOS and commercial antifungals, in the inhibition of medically relevant yeasts. Thus, we addressed the potential of CHOS for use in combination therapy.

## Materials and methods

### Enzymatic production of CHOS

Chitosan (KitoNor, FA 0.15, DPn 206) was obtained from Norwegian Chitosan, Gardermoen, Norway. CHOS were produced by enzymatic hydrolysis of the chitosan by chitosanase ScCsn46A and the resulting CHOS (abbreviated C32) were determined to be of CHOS with DP_n_ of 32 and *F*_A_ of 0.15 as described previously [22].

For further fractionation, C32 was dissolved in water to a concentration of 20 mg/mL and dialyzed against distilled water using Spectra/Por 6 dialysis membranes with cutoffs of 3.5 kDa, 8.0 kDa, 10 kDa, or 15 kDa, (Spectrumlabs, Rancho Dominguez, CA, USA). Each dialysis step was performed at 4°C with stirring for 48h. At the end of each dialysis step, the retentate and/or permeate was collected and lyophilized. Prior to use in biological experiments, the CHOS-powder was dissolved in two-fold concentrated culture medium and sterilized by filtration [21].

### Determination of the average degree of polymerization (DPn) with ^1^H-NMR spectroscopy

^1^H NMR experiments were performed on an Avance^TM^ 400 instrument from Bruker. The DP_n_ was calculated by the equation (Dα+Dβ+D+Aα+Aβ+A)/(Dα+Dβ+Aα+Aβ), where Dα, Dβ, Aα and Aβ are the integrals of the reducing end signals of the α and β anomers of the deacetylated (GlcN, D) and acetylated (GlcNAc, A) units, D is the integral of the signals from the internal and non-reducing end deacetylated units and A is the integral of the signals from the internal and non-reducing end acetylated units [23].

### Determination of relative molecular weights of CHOS fractions

Size exclusion chromatography was performed on a Dionex Ultimate 3000RSLC system (ThermoScientific, Sunnyvale USA) with RI detection. The columns were TOSOH TSKgel G3000PWXL-CP (7.8 x 300 mm, 7 μm) and TOSOH TSKgel G-oligoPW (7.8 x3 00 mm, 7 μm) coupled in series and operated isocratically at 1 mL/min with 0.1 M NaNO_3_ as the mobile phase. Samples were dissolved in the mobile phase. The system was calibrated with DIN-pullulan standards with molecular masses of 6 kDa, 12 kDa, 22 kDa, 50 kDa and 110 kDa (PSS Polymer Standards Service, Mainz, Germany). Chromatography data were exported and treated by WinGPC Scientific v 6.20 software for estimation of average molecular weights, degree of polymerization, average molecular mass, and dispersity [21].

### Antifungals

Commercial antifungals (CA), fluconazole (Flu), amphotericin B (Amp), voriconazole (Vor), flucytosine (Fcs), and miconazole (Mcz), were purchased from Sigma (St. Louis, MO).

### Yeast strains

Growth inhibition tests were performed using *Candida parapsilosis* 220919, *Candida tropicalis* 13803, and *Candida norvegensis* 22977 strains from the American Type Culture Collection (ATCC). Additionally, the clinical isolates *Candida albicans* (1581), *Candida guillermondii* (12146), *Candida lusitaneae* (20949), *Candida glabrata* (3808), *Rhodotorula*. *glutinis* (909700100), and *Rhodotorula mucilaginosa* (37016), belonging to the Oslo University Hospital collection were used. The strains were kept frozen in YPD broth at -70°C until testing.

### Preparation of inocula

Yeast strains were cultured in Sabouraud agar and incubated for 48 h at 37°C. Yeast suspensions were prepared in sterile water by touching ten colonies from a culture plate and adjusting the resulting suspension to 0.5 McFarland turbidity standard (approximately 5.5 x 10^6^ CFU mL^-1^) using spectrophotometric methods. One milliliter of the fungal suspension was added to 9 mL of RPMI (pH 6), providing the starting inoculum of approximately 5.5 x 10^5^ CFU mL^-1^.

### Analysis of synergistic effects

Checkerboard synergy testing was performed in triplicate using combinations of CHOS and CA as follows. Briefly, 100 μL of yeast inoculum obtained as previously described were added to a 96-well microplate containing different combinations of C32 and CA in potato dextrose agar (PDA) to a total volume of 200 μL, yielding final concentrations of 4.9 to 5000 μg mL^-1^ (CHOS) and 0.01 to 64 μg mL^-1^ (CA). Positive growth controls were performed in wells not containing antifungals. The minimal inhibitory concentration (MIC) was defined as the lowest drug concentration at which there was no visible growth after 48 h incubation at 37°C. The minimal inhibitory concentration in combination (MICC) was the lowest concentration of the drug and CHOS, respectively, when used in combination at which there was no visible growth after 24 h or 48 h incubation at 37°C. To evaluate the effect of the combinations, the fractional inhibitory concentration (FIC) was calculated for each antifungal (i.e. CHOS and CA) in each combination. The following formulas were used to calculate the FIC index: FIC of antifungal A equals MICC of A divided by MIC of A; FIC of antifungal B equals MICC of B divided by MIC of B; and FIC index equals FIC of antifungal A + FIC of antifungal B. Synergy was defined as an FIC index of ≤ 0.5 Indifference was defined as an FIC index of > 0.5 and ≤ 4. Antagonism was defined as an FIC index of > 4 [24]. Time-kill curves were determined incubating Flu and Amp at concentrations at 0.25 x MIC or MICC, separately, in the presence of C32 or absence of C32 (control), for a period of 48 h at 37°C. Samples were taken at different times and cultured on Sabouraud agar and incubated at 37°C for 48 h.

### Sequential therapy

Inocula obtained as previously described were incubated in the absence (control) and presence of C32 (0.25 x MIC) for 12 h at 37°C. The cells were then washed three times, and cell suspensions were adjusted to 0.5 McFarland turbidity standard in PDA and inoculated into flasks containing Flu or Amp (0.25 x MIC). Samples were taken at regular intervals to record survival. Numbers of living cells were determined by incubation on Sabouraud agar at 37°C for 48 h.

### Post-antifungal effects

Continuous antifungal effect (CAFE) and post-antifungal effect (PAFE) were determined *in vitro* as follows. Yeast inocula were exposed to 0.5 x MIC, 1.0 x MIC, and 2.0 x MIC of C32 for 2h at 37°C. For the control samples, no C32 was used. Afterwards, cells were collected by centrifugation, washed twice with PDA, and resuspended in fresh medium containing no anti-fungals. At different times, samples were taken, plated on Sabouraud agar and incubated for 48 h at 37°C. The PAFE was calculated using the formula PAFE = T-C, where T is the time required for the titer to increase 1 log10 over the post-washing titer for cells grown in the presence of C32, and C is the time required for the titer to increase 1 log10 over the post-washing titer for cells grown the absence of C32. For determining of the CAFE, yeasts were grown in the presence of 0.5 x MIC, 1.0 x MIC, and 2.0 x MIC of C32 for 24 h at 37°C. Samples were taken at different times during this 24h period and plated on Sabouraud agar. Calculations were made using the formula CAFE = T—C, where T is the time required for the titer to increase 1 log10 in the presence of C32, and C was the time required for the titer to increase 1 log10 in the absence of C32.

### Statistical analysis

Experiments were done in triplicate. Experimental data was analyzed using Minitab version 16 (Minitab 16, State College, PA). Student’s *t-*tests were performed to identify differences between samples. Differences were considered to be significant when *p* ≤ 0.05. Tukey's range test was used to assess differences between pairs of means.

## Results

In this study, the *in vitro* antifungal activity of C32, a chito-oligosaccharide mixture with DP_n_ 32 and F_A_ 0.15, was analyzed against clinical-relevant yeast strains, and the effect of the CHOS preparation was also determined when combined with five commercial antifungals: amphotericin B (Amp), fluconazole (Flu), voriconazole (Vor), flucytosine (Fcs), and miconazole (Mcz) (Table 1). The MIC of C32 varied greatly among strains, ranging from > 5000 μg mL^-1^ (*C*. *albicans* and *C*. *glabrata*) to < 4.9 μg mL^-1^ (*C*. *tropicalis*). Inhibitory effect of the CAs also varied among strains. In the case of Amp, the MIC ranged from 0.25 μg mL^-1^ (*C*. *guillermondii* and *C*. *norvegensis*) to 32 μg mL^-1^ (*C*. *lusitaneae*), whilst for Flu the values ranged from 4 μg mL^-1^ (*C*. *tropicalis*) to >64 μg mL^-1^ (*C*. *norvegensis*). The MIC was between 0.12 μg mL^-1^ (*C*. *albicans*) and 4 μg mL^-1^ (*C*. *lusitaneae*) for Vor and Fcs, and for Mcz, the MIC was >16 μg mL^-1^ for both tested yeasts, *C*. *albicans* and *C*. *glabrata*.

**Table 1:** Combined effect of C32 and CA on the growth of clinically relevant yeasts after 24 h of incubation. MIC: minimum inhibitory concentration; MICC: minimum inhibitory concentration in combination (= MIC of the compound in the presence of the other compound); S: Synergy; I: Indifference. All values are in μg mL^-1^.

	CA	MIC CA	MICC CA	MIC C32	MICC C32	Interaction
*C*. *albicans*				>5000		
	Amp	0.50	0.60		156.3	S
	Flu	64.0	0.50		19.5	S
	Vor	0.12	0.06		39..1	S
	Fcs	0.12	0.06		312.5	S
	Mcz	>16.0	0.50		4.9	S
*C*. *guillermondii*				39.1		
	Amp	0.25	0.01		<4.9	S
	Flu	32.0	0.01		<4.9	S
	Vor	1.0	0.06		<4.9	S
	Fcs	1.0	0.06		<4.9	S
*C*. *glabrata*				>5000		
	Flu	>32.0	32.0		5000	I
	Mcz	>16.0	1.0		156.3	S
*C*. *parapsilosis*				4.9		
	Amp	1.0	0.25		<4.9	I
	Flu	32.0	4.0		<4.9	S
	Vor	0.50	0.25		<4.9	I
	Fcs	0.50	0.06		<4.9	S
*C*. *tropicalis*				4.9		
	Amp	1.0	0.13		4.9	I
	Flu	4.0	1.0		4.9	I
*C*. *norvegensis*				9.8		
	Amp	0.25	0.03		<4.9	S
	Flu	>64	8.0		<4.9	S
*C*. *lusitaneae*				78.1		
	Amp	32.0			9.8	S
	Flu	32.0			19.5	S
	Vor	4.0			<4.9	S
	Fcs	4.0			<4.9	S
*R*. *glutinis*				78.1		
	Amp	1.0	0.06		9.8	S
	Flu	>64.0	16.0		9.8	S
	Vor	2.0	0.06		<4.9	S
	Fcs	2.0	0.06		<4.9	S
*R*. *mucilaginosa*				4.9		
	Amp	0.50	0.13		<4.9	S
	Flu	>64.0	>64.0		<4.9	I
	Vor	0.50	0.50		4.9	I
	Fcs	0.50	0.06		<4.9	S

Table 1 further shows that in most cases the combination of C32 and CA had clear synergistic effects and that some observed synergies were very strong. For example, the MIC for C32 and Mcz acting on *C*. *albicans* were reduced from >5000 μg mL^-1^ to 4.9 μg mL^-1^ and >16 μg mL^-1^ to 0.5 μg mL^-1^, respectively, when combined.

**Fig 1:**
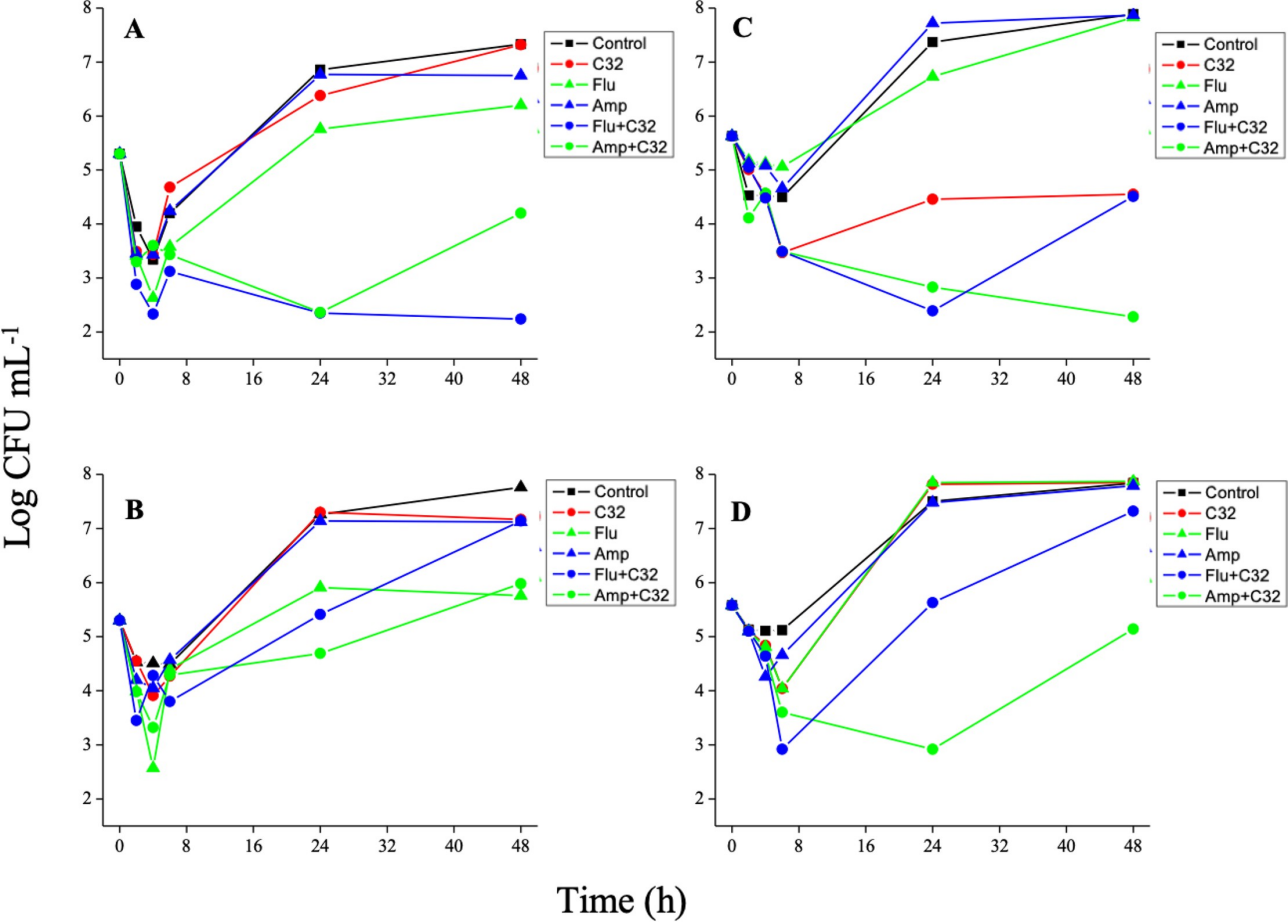
Inhibition of *C*. *albicans* (a, b) and *C*. *guillermondii* (c, d) when exposed to sub-inhibitory concentrations of CA and C32 at 0.25 x MIC (a, c) or MICC (b, d). The CA were applied at MICC, i.e. at concentrations that hardly inhibit the yeasts. The graphs show the amount of viable cells over time. Color coding is provided in the Figure. Standard deviations are omitted for clarity. Normally, these were between 0.1 to 0.5 Log CFU ml^-1^ units.

A time-kill study was performed on *C*. *albicans* and *C*. *guillermondii*, in order to analyze the combined effect of C32 and Flu or Amp in more detail (Fig 1). When using CA concentrations that only weakly inhibited growth of *C*. *albicans*, the combination with C32 at 0.25 MIC yielded a dramatic decrease in cell viability (Fig 1A), the enhancing effect being higher for Flu than for Amp. A similar, but less pronounced effect was observed after combining the CA with C32 at its MICC (Fig 1B). Similar results were obtained for the C32-sensitive strain *C*. *guillermondii*. In both cases we observed that the combination of C32 and Amp or Flu reduced yeast growth to a higher extent than what one would expect based on the sum of the reductions caused by each individual antifungal.

**Fig 2:**
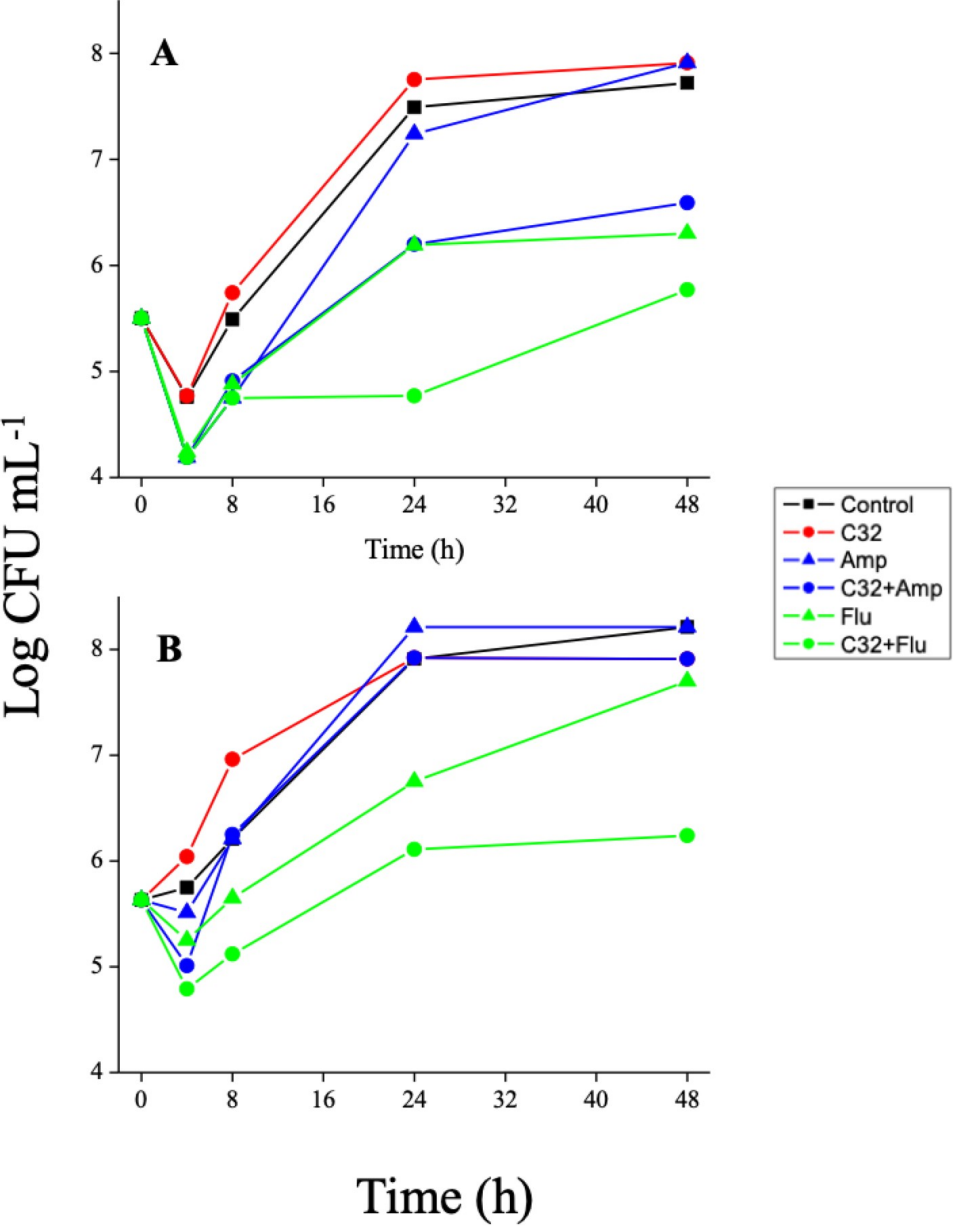
Sequential therapy time-kill curve for *C*. *albicans* (a) and *C*. *guillermondii* (b) in the presence of amphotericin B or fluconazole (concentration = 0.25 x MIC) after 12 h pre exposure to C32 (concentration = 0.25 x MIC). Color coding is provided in the Figure. Standard deviations are omitted for clarity. Normally, these were between 0.1 to 0.5 Log CFU ml^-1^ units.

The effect of Flu and Amp on yeasts previously exposed to C32 (0.25 x MIC) for 12 h was studied (Fig 2). Pre exposure to C32 enhanced the antifungal effect of Flu and Amp even after 24 h incubation. Growth curves of *C*. *guillermondii* pre-exposed to various concentrations of C32 (0.5, 1.0, and 2.0 x MIC) further showed a concentration-dependent post-exposure antifungal effect, even after 24 h. Harmonizing with these results, PAFE values (Table 2) showed that a certain degree of antifungal activity was maintained after removal of C32. However, the activity was significantly (*p* ≤ 0.05) lower than before C32 removal (CAFE).

**Table 2:** PAFE and CAFE for *C*. *guillermondii* in the presence of 0.5, 1.0 and 2.0 x MIC C32. Results expressed as average ± standard deviation. Results are expressed in time (h). Values that do not share the same letter are significantly different.

Concentration (x MIC)	CAFE	PAFE
0.5	2.52 ± 0.06	0.23 ± 0.11
1.0	4.20 ± 0.25	0.42 ± 0.44
2.0	6.06 ± 0.36	0.74 ± 0.03

To assess the size-dependency of CHOS with respect to antifungal and synergistic effects, the C32 preparation was fractionated using dialysis [21]. Four new mixtures were prepared: i) below 3.5 kDa, ii) between 3.5 kDa and 8.0 kDa, iii) above 3.5 kDa, and iv) above 10 kDa. Table 3 shows the properties of the various preparations, including the average degree of polymerization (DP_n_) was determined by ^1^H NMR [23] and sample naming. Table 3 also shows the relative molecular weight average (MW) estimated from analytical size-exclusion chromatography using pullulan standards with molecular weights of 6 kDa, 12 kDa, 22 kDa, 50 kDa, and 110 kDa (Table 3). The DP_n_ values of the obtained four fractions were 17, 31, 54 and 62.

**Table 3:** Molecular size determination of chitosan fractions by 1H-NMR and SEC.

Fraction	C32	C17	C31	C54	C62
cutoff (kDa)^a^	n.a.	<3.5	>3.5<8	>3.5	>10
1H-NMR (DP_n_)^b^	32	17	31	54	62
MW (kDa)^c^	15	7.6	15	21	24

^a^ Dialysis cut-offs used to separate hydrolyzed chitosan into different CHOS preparations.

^b^ Standard NMR method used to characterize CHOS fractions. These DP_n_ values are used to name the CHOS fractions.

The inhibitory effects of the four CHOS mixtures were studied *in vitro* on 4 clinically relevant yeast strains (Table 4), using two CAs. The data show a clear size dependency both for the inhibitory effect of CHOS alone and the overall impact and synergistic effects of combining CHOS and CA. The C31 fraction and, to a slightly lesser extent, the C54 fraction stand out as superior, relative to the C17 and C62 fractions.

**Table 4:** Combined effect of CA and CHOS with different DPn on the growth of yeast strains. CA: commercial antifungals, MIC: minimum inhibitory concentration, MICC: minimum inhibitory concentration in combination when administered simultaneously, Flu: fluconazole, Amp: amphotericin B. Data expressed in μg mL^-1^.

CA	CHOS	MIC CA		MICC CA	MIC CHOS	MICC CHOS
*C*. *albicans*						
Amp			0.5			
	C17			0.06	>5000	156.3
	C31			0.06	>5000	156.3
	C54			0.06	>5000	156.3
	C62			0.06	>5000	2500
Flu		>16.0				
	C17			2.0	>5000	156.3
	C31			0.50	>5000	19.5
	C54			0.50	>5000	19.5
	C62			4.0	>5000	2500
*C*. *guillermondii*						
Amp		0.25				
	C17			0.06	78.1	19.5
	C31			0.01	39.1	<4.9
	C54			0.03	39.1	<4.9
	C62			0.12	>312.5	156.3
Flu		>16.0				
	C17			0.06	78.1	9.8
	C31			0.01	39.1	<4.9
	C54			0.03	39.1	<4.9
	C62			0.12	>312.5	39.1
*C*. *lusitaneae*						
Amp		0.50				
	C17			4.0	156.3	78.1
	C31			1.0	78.1	9.8
	C54			2.0	78.1	19.5
	C62			4.0	>312.5	19.5
Flu		>16.0				
	C17			4.0	156.3	78.1
	C31			1.0	78.1	9.8
	C54			2.0	78.1	19.5
	C62			4.0	>312.5	19.5
*C*. *parasilopsis*						
Amp		1.0				
	C17			0.25	19.5	9.8
	C31			0.50	4.9	<4.9
	C54			0.25	19.5	9.8
	C62			0.25	>312.5	156.3
Flu		>16.0				
	C17			16.0	19.5	9.8
	C31			8.0	4.9	<4.9
	C54			16.0	39.1	9.8
	C62			16.0	>312.5	156.3

## Discussion

Combined antifungal therapies have received great interest due to their potential of overcoming fungal resistance to conventional treatments. In this study, the feasibility of using a combined therapy of a well-characterized CHOS (C32) and five well-established CA against medically relevant yeasts strains was analyzed *in vitro*. We show that combining C32 with CA yield synergistic effects. The magnitude of these effects differs between yeasts and CA: in some cases, the synergistic effects seemed very strong.

Some reports have suggested that the polycationic character of chitosan is responsible for its antifungal activity, since these cationic groups may interact with anionic components of the cell wall of the fungi and destabilize their membrane [21, 25, 26]. It thus seems reasonable to hypothesize that increased membrane permeability promoted by C32 might allow the CA to penetrate the target cells more easily. In the case of the azoles tested (Flu and Vor), this increased CA flux into the cell cytosol might increase the inhibition of the production of 14α-demethylase resulting in reduction of the membrane fluidity and an increase in the production of toxic sterols [27, 28]. Likewise, for Fcs, increased membrane permeability could improve its flux into the cytosol where Fcs is suggested to interact with RNA biosynthesis [29]. For Amp, one may speculate that alterations in the cell membrane caused by C32 contribute in a synergistic way to the membrane destabilization caused by the formation of Amp-driven transmembrane channels, leading to the collapse and death of the cell [30].

Interestingly, synergy was observed even in the case of *C*. *albicans*, for which C32 was not effective when used alone. This result suggests that the CHOS might, to some extent, be disturbing the cell membrane, even if it does not affect the cell viability. Similar results were obtained in a previous study conducted by Palmeira-de-Oliveira *et al*. [31]. Through a cytometric analysis the authors showed that a chitosan hydrogel induced primary lesions on the cell membrane of *Candida* spp. even under conditions that did not reduce cell viability.

The present results agree with those reported by Jaime *et al*. [27] on *Saccharomyces cerevisiae*, who found that the combination of CHOS (84 μg mL^-1^, 5.44 kDa and 97% degree of deacetylation) and Flu (20 μg mL^-1^) had a synergistic effects on growth inhibition. Contrarily, in a different study Calamari *et al*. [32] studied the activity of fluconazole (50-100-150 μg mL^-1^), chitosan (0.25%, 300 kDa, 90% F_A_), chlorhexidine (12.5-25-50 μg mL^-1^) and their combinations against *C*. *albicans* and observed no synergistic effects. Additionally, in a different study, no apparent synergistic activity between commercial available chitosan (Mw = 70 kDa and Fa = 0.25) and fluconazole was reported on clinical *Candida* strains [18]. These results combined with those obtained in the present study suggest that both DP_n_ and Fa are important to obtain synergy with CAs.

Increase in antifungal activity of a compound due to the presence of chitosan has been observed previously. For instance, a chitosan gel was shown to increase the antifungal activity of a membrane-destabilizer chlorhexidine-gluconate [33]. Additionally, the combination of chitosan acetate (ChA) and another membrane-disrupting compound (EDTA) showed a synergistic effect on *C*. *albicans*. El-Sharif and Hussain reported a dramatic reduction in MIC values when using chitosan acetate and EDTA in combination (MICC ChA = 0.5 μg mL^-1^, MICC EDTA = 0.5 μg mL^-1^) compared the individual compounds (EDTA = 850 μg/ml, ChA = 500 μg mL^-1^) [34].

The present study shows that synergistic effects between CHOS and CA are strongest for the C31 and C54 fraction. In a previous study, Rahman *et al*. [22] studied the effect of CHOS with different DP_n_ on germination of *Botrytris cinerea* and *Mucor piriformis*. The authors found that CHOS with DP_n_ 23 and 40 had the strongest inhibitory effect against the tested pathogens. The original CHOS (DP_n_ 206) and shorter CHOS were considerably less effective. Thus, accumulating data indicate that CHOS with a DP_n_ near 30 are of particular interest for application as anti-fungals. As discussed above, it is believed that CHOS adsorbs to the cell surface, disturbs membrane integrity, and may accumulate inside the cells. Our results indicate that this membrane disturbance depends on the length of the CHOS. Although C17 and C62 show inhibitory effects (MIC, Table 4) on most of the strains and clearly display synergies with commercial antifungals on all strains (MICC, Table 4), the effects are less pronounced than what is observed for i.e. C31 and C54.

Additionally to combined treatments, sequential therapy has been intensively studied as an alternative for fighting resistant yeast infections [35]. The present study shows that pre exposure to C32 enhances the inhibitory effects of subsequent administration of Flu and Amp, even 24 h after C32 removal. Combined and sequential antifungal therapy outcomes are related to the presence of a PAFE, which is a term used to describe the persistent suppression of fungal growth after limited exposure to an antifungal. This feature is useful for the evaluation of the pharmacokinetic and pharmacodynamic indices, which are closely associated with the efficacy of the antifungal agents *in vivo*. In this sense, it can be expected that antifungals with long PAFE may be administered less frequently than those with short ones, which may require more frequent administration [36, 37]. A common assumption is that the PAFE is the result of the inhibition of microbial growth with a consequent prolongation of the lag time. However, antifungals with long PAFE are capable of exerting many different effects on surviving fungi, detectable after the drug has been removed, including prolonged changes in cell morphology, metabolism, growth and generation time as well as delayed protein synthesis and altered susceptibility to other antifungals [38].

Recently, Wang *et al*. [39] studied the post-antifungal effect of a chitosan/nano-ZnO nanofibrous membrane on *C*. *albicans* and reported concentration-dependent PAFE of 4.1 ± 0.2 h, 8.2 ± 0.2 h, 10.2 ± 0.2 h, for 0.5 x MIC; 1.0 x MIC and 1.5 x MIC, respectively. Additionally, several studies have evaluated the PAFE of commercial drugs. For instance, Ernst *et al*. [36] reported Flu concentration-dependent PAFE for *C*. *albicans* that ranged between 4 h and >12 h (0.25 x MIC). Also, Manavathu *et al*. [40] reported a PAFE for Amp (μg mL^-1^) on *C*. *albicans* of 5.3 ± 1.15 h, while Egusa *et al*. [41] reported that the mean duration of PAFE of amphotericin for *C*. *albicans* was 8.73 ± 0.93 h (2 x MIC). Interestingly, PAFE values obtained in our study were considerably lower, in spite of C32 maintaining its positive effect of the efficacy of some CAs for up to 48 h after removal. This observation reinforces our theory of C32 causing a perturbation of the cell membrane without causing altering of the growth rate.

The combination of CHOS and conventional antifungals, together or as part of a sequential therapy, opens new therapeutic perspectives for treating human candidiasis. The synergistic effects described above may be useful to reduce antifungal dosages without substantially compromising the efficacy, to broaden the spectrum of anti-fungal activity, and/or to improve the efficacy of current antifungals. Overcoming the rising resistance of yeasts to current treatments is another perspective of the results described above.

## References

[1] Roling EE, Klepser ME, Wasson A, Lewis RE, Ernst EJ, Pfaller MA. Antifungal activities of fluconazole, caspofungin (MK0991), and anidulafungin (LY 303366) alone and in combination against Candida spp. and Crytococcus neoformans via time-kill methods. Diagn Microbiol Infect Dis. 2002;43(1):13–7. 10.1016/s0732-8893(02)00361-9 12052624

[2] Louie A, Kaw P, Banerjee P, Liu W, Chen G, Miller MH. Impact of the order of initiation of fluconazole and amphotericin B in sequential or combination therapy on killing of Candida albicans in vitro and in a rabbit model of endocarditis and pyelonephritis. Antimicrob Agents Chemother. 2001;45(2):485–94. Epub 2001/02/13. 10.1128/AAC.45.2.485-494.2001 11158745 PMC90317

[3] Baddley J, Poppas P. Antifungal Combination Therapy. Drugs. 2005;65(11):1461–80. 10.2165/00003495-200565110-00002 16033288

[4] Uppuluri P, Nett J, Heitman J, Andes D. Synergistic Effect of Calcineurin Inhibitors and Fluconazole against Candida albicans Biofilms. Antimicrob Agents Chemother. 2008;52(3):1127–32. 10.1128/AAC.01397-07 18180354 PMC2258509

[5] Healey KR, Perlin DS. Fungal Resistance to Echinocandins and the MDR Phenomenon in Candida glabrata. J Fungi. 2018;4(3):105 10.3390/jof4030105 . 30200517 PMC6162769

[6] Ben-Ami R. Treatment of Invasive Candidiasis: A Narrative Review. J Fungi. 2018;4(3):97 10.3390/jof4030097 . 30115843 PMC6162658

[7] Pristov KE, Ghannoum MA. Resistance of Candida to azoles and echinocandins worldwide. Clin Microbiol Infect. 2019;25(7):792–8. 10.1016/j.cmi.2019.03.028 30965100

[8] Beer KD, Farnon EC, Jain S, Jamerson C, Lineberger S, Miller J, et al Multidrug-Resistant Aspergillus fumigatus Carrying Mutations Linked to Environmental Fungicide Exposure—Three States, 2010–2017. MMWR. 2018;67(38):1064–7. 10.15585/mmwr.mm6738a5 . 30260939 PMC6188124

[9] Verweij PE, Chowdhary A, Melchers WJG, Meis JF. Azole Resistance in Aspergillus fumigatus: Can We Retain the Clinical Use of Mold-Active Antifungal Azoles? Clin infect Dis. 2016;62(3):362–8. Epub 2015/10/20. 10.1093/cid/civ885 . 26486705 PMC4706635

[10] Baddley JW, Stroud TP, Salzman D, Pappas PG. Invasive mold infections in allogeneic bone marrow transplant recipients. Clin Infect Dis. 2001;32(9):1319–24. Epub 2001/04/17. CID000656 [pii] 10.1086/319985 . 11303267

[11] Cortez KJ, Roilides E, Quiroz-Telles F, Meletiadis J, Antachopoulos C, Knudsen T, et al Infections Caused by Scedosporium spp. Clinical Microbiology Reviews. 2008;21(1):157–97. 10.1128/CMR.00039-07 18202441 PMC2223844

[12] Aam BB, Heggset EB, Norberg AL, Sørlie M, Vårum KM, Eijsink VGH. Production of chitooligosaccharides and their potential applications in medicine. Marine Drugs. 2010;8:1482–517. 10.3390/md8051482 20559485 PMC2885077

[13] Rinaudo M. Chitin and chitosan: Properties and applications. Prog Polym Sci. 2006;31(7):603–32.

[14] Singh K, Tiwary AK, Rana V. Spray dried chitosan-EDTA superior microparticles as solid substrate for the oral delivery of amphotericin B. International Journal of Biological Macromolecules. 2013;58:310–9. 10.1016/j.ijbiomac.2013.04.053 23624284

[15] Tiyaboonchai W, Limpeanchob N. Formulation and characterization of amphotericin B-chitosan-dextran sulfate nanoparticles. Int J Pharm. 2007;329(1–2):142–9. Epub 2006/09/27. S0378-5173(06)00660-0 [pii] 10.1016/j.ijpharm.2006.08.013 . 17000065

[16] Gratieri T, Gelfuso GM, de Freitas O, Rocha EM, Lopez RF. Enhancing and sustaining the topical ocular delivery of fluconazole using chitosan solution and poloxamer/chitosan in situ forming gel. Eur J Pharm Biopharm. 2011;79(2):320–7. Epub 2011/06/07. S0939-6411(11)00166-4 [pii] 10.1016/j.ejpb.2011.05.006 . 21641994

[17] Yehia SA, El-Gazayerly ON, Basalious EB. Fluconazole mucoadhesive buccal films: in vitro/in vivo performance. Curr Drug Deliv. 2009;6(1):17–27. Epub 2009/05/08. 10.2174/156720109787048195 . 19418952

[18] Alburquenque C, Bucarey SA, Neira-Carrillo A, Urzua B, Hermosilla G, Tapia CV. Antifungal activity of low molecular weight chitosan against clinical isolates of Candida spp. Medical mycology. 2010;48(8):1018–23. Epub 2010/05/21. 10.3109/13693786.2010.486412 . 20482450

[19] Martinez LR, Mihu MR, Tar M, Cordero RJ, Han G, Friedman AJ, et al Demonstration of antibiofilm and antifungal efficacy of chitosan against candidal biofilms, using an in vivo central venous catheter model. J Infect Dis. 2010;201(9):1436–40. Epub 2010/03/25. 10.1086/651558 . 20331379

[20] Palmeira-de-Oliveira A, Ribeiro MP, Palmeira-de-Oliveira R, Gaspar C, Costa-de-Oliveira S, Correia IJ, et al Anti-Candida activity of a chitosan hydrogel: mechanism of action and cytotoxicity profile. Gynecol Obstet Invest. 2010;70(4):322–7. Epub 2010/11/27. 000314023 [pii] 10.1159/000314023 . 21109742

[21] Ganan M, Lorentzen SB, Agger JW, Heyward CA, Bakke O, Knutsen SH, et al Antifungal activity of well-defined chito-oligosaccharide preparations against medically relevant yeasts. PLOS ONE. 2019;14(1):e0210208 10.1371/journal.pone.0210208 30620751 PMC6324834

[22] Rahman MH, Shovan LR, Hjeljord LG, Aam BB, Eijsink VGH, Sørlie M, et al Inhibition of Fungal Plant Pathogens by Synergistic Action of Chito-Oligosaccharides and Commercially Available Fungicides. Plos One. 2014;9(4):10 10.1371/journal.pone.0093192 WOS:000336736600002. 24770723 PMC4000203

[23] Sørbotten A, Horn SJ, Eijsink VGH, Vårum KM. Degradation of chitosans with chitinase B from *Serratia marcescens*. Production of chito-oligosaccharides and insight into enzyme processivity. Febs J. 2005;272(2):538–49. 10.1111/j.1742-4658.2004.04495.x 15654891

[24] White RL, Burgess DS, Manduru M, Bosso JA. Comparison of three different in vitro methods of detecting synergy: time-kill, checkerboard, and E test. Antimicrob Agents Chemother. 1996;40(8):1914–8. 8843303 10.1128/aac.40.8.1914 PMC163439

[25] Park Y, Kim MH, Park SC, Cheong H, Jang MK, Nah JW, et al Investigation of the antifungal activity and mechanism of action of LMWS-chitosan. Journal of microbiology and biotechnology. 2008;18(10):1729–34. Epub 2008/10/29. . 18955827

[26] Rabea EI, Badawy ME, Stevens CV, Smagghe G, Steurbaut W. Chitosan as antimicrobial agent: applications and mode of action. Biomacromolecules. 2003;4(6):1457–65. Epub 2003/11/11. 10.1021/bm034130m . 14606868

[27] Jaime MDLA, Lopez-Llorca LV, Conesa A, Lee AY, Proctor M, Heisler LE, et al Identification of yeast genes that confer resistance to chitosan oligosaccharide (COS) using chemogenomics. BMC Genomics. 2012;13: 267 10.1186/1471-2164-13-267 22727066 PMC3505485

[28] Lamb DC, Kelly DE, Schunck W-H, Shyadehi AZ, Akhtar M, Lowe DJ, et al The Mutation T315A in Candida albicans Sterol 14α-Demethylase Causes Reduced Enzyme Activity and Fluconazole Resistance through Reduced Affinity. J Biol Chem. 1997;ß(9):5682–8. 10.1074/jbc.272.9.5682 9038178

[29] Vermes A, Guchelaar HJ, Dankert J. Flucytosine: a review of its pharmacology, clinical indications, pharmacokinetics, toxicity and drug interactions. J Antimicrob Chemother. 2000;46(2):171–9. Epub 2000/08/10. 10.1093/jac/46.2.171 . 10933638

[30] HR W. Mechanism of action of antieukaryotic and antiviral compounds. G D., SP D., editors. New York: Springer; 1979.

[31] Palmeira-de-Oliveira A, Ribeiro MP, Palmeira-de-Oliveira R, Gaspar C, Costa-de-Oliveira S, Correia IJ, et al Anti-Candida activity of a chitosan hydrogel: mechanism of action and cytotoxicity profile. Gynecologic and Obstetric Investigation. 2010;70(4):322–7. 10.1159/000314023 21109742

[32] Calamari SE, Bojanich MA, Barembaum SR, Berdicevski N, Azcurra AI. Antifungal and post-antifungal effects of chlorhexidine, fluconazole, chitosan and its combinations on Candida albicans. Medicina oral, patologia oral y cirugia bucal. 2011;16(1):e23–8. Epub 2010/08/17. 10.4317/medoral.16.e23 . 20711160

[33] Senel S, Ikinci G, Kas S, Yousefi-Rad A, Sargon MF, Hincal AA. Chitosan films and hydrogels of chlorhexidine gluconate for oral mucosal delivery. Int J Pharm. 2000;193(2):197–203. Epub 1999/12/22. S0378-5173(99)00334-8 [pii]. 10.1016/s0378-5173(99)00334-8 . 10606782

[34] El-Sharif AA, Hussain MH. Chitosan-EDTA new combination is a promising candidate for treatment of bacterial and fungal infections. Curr Microbiol. 2010;62(3):739–45. Epub 2010/10/22. 10.1007/s00284-010-9777-0 . 20963418

[35] Kontoyiannis DP, Lewis RE. Combination chemotherapy for invasive fungal infections: what laboratory and clinical studies tell us so far. Drug Resist Updates. 2003;6(5):257–69. 10.1016/j.drup.2003.08.003. 14643296

[36] Ernst EJ, Klepser ME, Pfaller MA. Postantifungal effects of echinocandin, azole, and polyene antifungal agents against Candida albicans and Cryptococcus neoformans. Antimicrob Agents Chemother. 2000;44(4):1108–11. Epub 2000/03/18. 10.1128/aac.44.4.1108-1111.2000 10722525 PMC89826

[37] D'Arrigo M, Ginestra G, Mandalari G, Furneri PM, Bisignano G. Synergism and postantibiotic effect of tobramycin and Melaleuca alternifolia (tea tree) oil against Staphylococcus aureus and Escherichia coli. Phytomed. 2010;17(5):317–22. Epub 2009/08/25. S0944-7113(09)00193-7 [pii] 10.1016/j.phymed.2009.07.008 . 19699074

[38] MacKenzie FM, Gould IM. The post-antibiotic effect. J Antimicrob Chemother. 1993;32(4):519–37. 10.1093/jac/32.4.519 8288494

[39] Wang Y, Zhang Q, Zhang C-l, Li P. Characterisation and cooperative antimicrobial properties of chitosan/nano-ZnO composite nanofibrous membranes. Food Chem. 2012;132(1):419–27. 10.1016/j.foodchem.2011.11.015 26434310

[40] Manavathu EK, Ramesh MS, Baskaran I, Ganesan LT, Chandrasekar PH. A comparative study of the post-antifungal effect (PAFE) of amphotericin B, triazoles and echinocandins on Aspergillus fumigatus and Candida albicans. J Antimicrob Chemother. 2004;53(2):386–9. Epub 2004/01/20. 10.1093/jac/dkh066 dkh066 [pii]. . 14729762

[41] Egusa H, Ellepola AN, Nikawa H, Hamada T, Samaranayake LP. Sub-therapeutic exposure to polyene antimycotics elicits a post-antifungal effect (PAFE) and depresses the cell surface hydrophobicity of oral Candida albicans isolates. J Oral Pathol Med. 2000;29(5):206–13. Epub 2000/05/09. 10.1034/j.1600-0714.2000.290503.x . 10801037

